# The Role of Proteinase-Activated Receptors 1 and 2 in the Regulation of Periodontal Tissue Metabolism and Disease

**DOI:** 10.1155/2017/5193572

**Published:** 2017-04-19

**Authors:** E. S. Rovai, M. Holzhausen

**Affiliations:** Division of Periodontics, Department of Stomatology, School of Dentistry, University of São Paulo, São Paulo, SP, Brazil

## Abstract

Proteinase-activated receptors 1 (PAR_1_) and 2 (PAR_2_) are the most highly expressed members of the PAR family in the periodontium. These receptors regulate periodontal inflammatory and repair processes through their activation by endogenous and bacterial enzymes. PAR_1_ is expressed by the periodontal cells such as human gingival fibroblasts, gingival epithelial cells, periodontal ligament cells, osteoblasts, and monocytic cells and can be activated by thrombin, matrix metalloproteinase 1 (MMP-1), MMP-13, fibrin, and gingipains from *Porphyromonas gingivalis*. PAR_2_ is expressed by neutrophils, osteoblasts, oral epithelial cells, and human gingival fibroblasts, and its possible activators in the periodontium are gingipains, neutrophil proteinase 3, and mast cell tryptase. The mechanisms through which PARs can respond to periodontal enzymes and result in appropriate immune responses have until recently been poorly understood. This review discusses recent findings that are beginning to identify a cardinal role for PAR_1_ and PAR_2_ on periodontal tissue metabolism.

## 1. Introduction

Periodontium is characterized by the tissues that involve and support the teeth such as the gingiva, alveolar mucosa, cementum, periodontal ligament, and alveolar bone. Periodontitis, an oral disease which leads to alveolar bone loss, can be mediated by some of the endogenous host enzymes, as well as bacterial proteinases present in the periodontal pocket (e.g., neutrophil serine proteinase 3), mast cell tryptase, and gingipain from *Porphyromonas gingivalis*. Interestingly, it was recently shown that the biological activities of these proteinases can be mediated through specific proteinase-activated receptor (PAR) activation. PARs are members of the G-protein-coupled family, seven-transmembrane domain receptors, and their activation occurs through proteolytic cleavage of the N-terminal domain by proteinases, leading to the generation of a new N-terminal “tethered ligand,” which binds to the receptor itself resulting in its autoactivation [1, 2]. Until now, four members of the PAR family were discovered: PAR_1_, PAR_3_, and PAR_4_ which are activated by thrombin and PAR_2_ that can be activated by trypsin, neutrophil proteinase 3, tissue factor/factor VIIa/factor Xa, mast cell tryptase, membrane-tethered serine proteinase-1, or gingipains [3, 4]. PARs represent a component of the innate inflammatory response, being involved in neutrophil recruitment, increased perfusion, pain, and swelling. Thus, since PARs are present in periodontal epithelial cells and are capable of recognizing and responding to bacterial infections, it is believed that they can act as a first “alarm system” for bacterial invasions [5]. In addition, studies have suggested an important role for PARs in regulating the inflammatory response intensity to bacterial infection, as well as in periodontitis [5, 6].

Although the structures and mechanisms related to the activation of these receptors are similar, they can be expressed by different cells; hence, in each cell, their activation may lead to distinct roles in pathophysiological processes, such as growth, development, inflammation, tissue repair, and pain [2, 7–10]. PAR_1_ is expressed by platelets, osteoblast endothelial cells, epithelial cells, fibroblasts, myocytes, neurons, and astrocytes, and it seems to play an important role in injured tissues. In the periodontium, the literature have also implicated PAR_1_ in bone repair and homeostasis [11–13], as well as proliferation of gingival fibroblasts mediated by the protein synthesis of endothelin-1 (ET-1) and subsequent activation of ET receptor type A [14] and transactivation of latent transforming growth factor beta 1 (TGF-*β*1) [15]. In addition, Rohani et al. [16] showed that in gingival epithelial cells, the PAR_1_ activation by thrombin can result in the induction of chemokines leading to granulocyte attraction. Interestingly, gingipain-R (RgpB and HRgpA), a proteinase from *Porphyromonas gingivalis*, can activate PAR_1_ in monocytic cells triggering an overproduction of proinflammatory cytokines [17] and in the surface of platelets leading to platelet aggregation [18]. This mechanism may constitute the biological plausibility of the association between periodontitis and cardiovascular disease and deserves further clarification by future studies.

PAR_3_ and PAR_4_ are expressed by platelets, endothelial cells, myocytes, and astrocytes, and their activation has been associated with the formation of pathologic thrombus [1]. The functional role of PAR_3_ is controversial, whereas some investigators have described it as a nonsignaling receptor that acts along with PAR_1_ and PAR_4_, others have claimed that PAR_3_ can signal independently of PAR_1_ activation. Thus, since PAR_3_ and PAR_4_ are less abundant than PAR_1_ in periodontal cells, and since their possible function on the periodontium still needs to be investigated [16], they are not going to be part of the present review.

PAR_2_ has been demonstrated to be expressed by epithelial cells, endothelial cells, fibroblasts, osteoblasts, myocytes, neurons, astrocytes, lymphocytes, neutrophils, and mast cells [1, 17, 19–21], where it has been associated to several roles in the inflammation process such as increased vascular permeability, blood vessel relaxation, hypotension, granulocyte infiltration, release of cytokines, and pain [3, 4, 17, 22–26]. Moreover, inflammatory events in the joints, skin, colon, kidney, and airways have also been associated to PAR_2_ activation [1, 2, 24, 27–29]. More recently, studies have shown that PAR_2_ activation may play an important role in the inflammatory process and tissue breakdown in periodontitis [6, 30–33].

In this review, we will discuss the possible roles of the most highly expressed members of the PAR family in the periodontium, PAR_1_ and PAR_2_, as important molecules that mediate mammalian and bacterial enzyme's effects on cells in the regulation of periodontal inflammation and repair.

## 2. Potential Activators of PARs and Their Inhibitors in the Periodontium

A nucleophilic Ser residue at the active site of serine proteinases gives this class of enzyme its name. These proteinases play important roles in several biological functions, like clot formation and wound healing through the ability to activate PARs. The main PAR-activating proteinases found in the periodontal environment are neutrophil proteinase 3, thrombin, plasmin, tryptase, MMP-1, MMP-13, and gingipains (Table 1).

Neutrophil proteinase 3 is a multifunctional serine proteinase mainly located on the cell surface and in the azurophilic granules of neutrophils which are the predominant cell type in the periodontal pocket of chronic periodontitis and represent the first line of defense against infection. Interestingly, in inflammatory states, the intracellular proteinase 3 can be translocated to the cell surface, thus increasing the accessibility of proteinase 3 to bind to molecules such as PARs [34]. As neutrophil proteinase 3, elastase is a proteinase stored in the secretory granules which are released during the inflammatory process. These neutrophil proteinases differentially activate PARs 1 and 2 by a biased signaling mechanism that can both disarm the receptors from thrombin and trypsin activation and can cleave the receptors at distinct N-terminal residues to unmask different “noncanonical” receptor-activating tethered ligand sequences, triggering different signaling pathways from thrombin and trypsin [2, 35, 36]. In PAR_2_, neutrophil proteinase 3, elastase, and cathepsin G (another neutrophil proteinase) can cleave the receptor downstream from the canonical trypsin site, serving as a deactivating proteinase and also as a biased PAR_2_ agonist. Although the impact of these proteinases in PAR_2_ activation is uncertain, it is believed that they play a role in inflammatory diseases [35].

Thrombin is another endogenous serine proteinase that can be released from fibrin clot following gingival tissue injury or inflammation. In gingival tissues, thrombin has been suggested to play a role in the healing processes, since it is known that thrombin-rich and platelet-rich plasma has been successfully used for periodontal regenerative surgery. Recently, it has been suggested that several cell functions that regulate inflammation, healing, and fibrosis in the periodontium can be mediated through the activation of PARs by thrombin [15].

Plasmin is a serine proteinase which not only acts on fibrin degradation leading to clot dissolution but also activates MMPs, growth factors, and proteinase-activated receptors (PAR_1_). Studies have shown that plasmin may activate different cell types, playing a role in the process of tissue remodeling, repair, and host defense [37–39]. In periodontium, Sulniute et al. [40] showed that plasmin plays an important role in preventing the development of chronic periodontitis in mice. In addition, a recent study suggested that plasmin may reduce lipopolysaccharide- (LPS-) induced inflammatory osteoclastogenesis through PAR_1_ activation [13]. Increased mast cell tryptase is found at the mucosal and subcutaneous connective tissue [41] and at the gingival crevicular fluid of patients with chronic periodontal diseases [42–45]. Holzhausen et al. [46] showed that the selective inhibition of tryptase with a compound named nafamostat mesilate leads to decreased gingival tissue granulocyte infiltration, decreased alveolar bone loss, and decreased PAR_2_ expression in the gingival tissue of rats subjected to experimental periodontitis, therefore suggesting that tryptase may play a role in the pathogenesis of chronic periodontal disease through PAR_2_ activation.

Interestingly, the zinc-dependent endopeptidases, MMP-1 and MMP-13, also have demonstrated the ability to activate PAR_1_. MMPs cleave PAR_1_ at noncanonical sites distinct from thrombin, generating unique tethered ligands which activate biased signaling pathways associated with thrombus initiation and thrombosis, atherosclerosis and restenosis, sepsis, angiogenesis, heart failure, and cancer. da Silva et al. [47] demonstrated that increased MMP-13 levels were associated with an increased PAR_1_ expression at the gingival crevicular fluid of patients with chronic periodontitis after nonsurgical periodontal treatment.

In addition to host origin proteinases, exogenous serine proteinases originated by periodontopathic bacteria can also play a role in the innate response mediated by PARs. *Porphyromonas gingivalis*, for instance, produces and releases the cysteine proteinases, arginine-gingipain (HRgpA and RgpB) and lysine-gingipain (Kgp) which are strongly associated with periodontal breakdown and disruption of host defense. Some of the mechanisms played by gingipains are mediated by PARs 1 and 2, due to their potential to interact with host cell surface receptors modulating the innate response.

Other nonmammalian proteinases produced by periodontal pathogens have already been suggested to play a role on PAR_2_ function. Dentilisin, a chymotrypsin-like enzyme produced by *Treponema denticola*, is suggested to cause PAR_2_ disarming or inhibition to further activation [6]. Interestingly, a study by Euzebio Alves et al. [32] has demonstrated an inverse relationship between PAR_2_ expression and the expression of dentilisin in the periodontal sites of moderate chronic periodontitis patients. Another bacterial proteinase, an arginine- and lysine-specific proteinase produced by *Aggregatibacter actinomycetemcomitans*, was shown to induce interleukin (IL)-8 and intercellular adhesion molecule-1 (ICAM-1) expression in gingival epithelial cells through PAR_2_ activation [48]. It can be suggested that bacterial proteinases produced by other periodontal pathogens could also play a role on the activation or suppression of PAR_2_ function or expression.

Interestingly, the plasma contains serine proteinase inhibitors (serpins) that can regulate proteolytic events in tissues [2, 49]. The easy accessibility of the reactive site loops of serpins guarantees the rapid inhibition of specific host proteinases, but it also makes them easy targets for bacterial proteinases, which can specifically inactivate them. In fact, the ability to resist inhibition by serpins is also important in host defense evasion by bacterial pathogens. Accordingly, Euzebio Alves et al. [32] have demonstrated that elevated levels of gingipain and proteinase 3 and decreased levels of secretory leucocyte proteinase inhibitor (SLPI) were associated to PAR_2_ overexpression. SLPI is expressed by epithelial and immune cells where they play a role as an alarm proteinase inhibitor mediating anti-inflammatory and antimicrobial effects. In this study, decreased levels of SLPI were found in chronic periodontitis patients, whereas periodontal treatment led to its upregulation. The authors suggested that these results might be explained by the ability of the arginine-specific gingipains (Rgps) to degrade SLPI. Similarly, reduced SLPI levels and higher serine proteinase activities correlating with PAR_2_ overexpression were found in the gastric mucosa of *Helicobacter pylori*-infected individuals. This fact may be associated to the loss of host protective capacity and increase susceptibility to breakdown from chronic infection. These data reinforce the role played by *Porphyromonas gingivalis* on PAR_2_-mediated periodontal inflammation.

## 3. Biological Effects of PAR_1_ Activation in Periodontal Cells and Tissues

PAR_1_ involvement in periodontal tissue metabolism has been suggested by several in vitro studies which have shown expression of its receptor by the periodontal cells such as human gingival fibroblasts, gingival epithelial cells, periodontal ligament cells, osteoblasts, and monocytic cells and presence of its possible activators, thrombin, plasmin, MMPs, and gingipains, in the periodontal environment (Table 2).

The biological effects of PAR_1_ activation on the periodontium are still not well clarified. Some studies have shown that PAR_1_ activation has a tissue destructive profile, leading to the induction of proinflammatory mediators that regulate periodontal breakdown, while others highlighted its possible involvement with the repair of periodontal tissues [11, 12].

Uehara et al. [50] have showed that production of hepatocyte growth factor (HGF) by human gingival fibroblasts upon stimulation with gingipains occurred through PARs, specifically PAR_1_ and PAR_2_. HGF plays a role in wound healing, through its mitogenic activity to gingival epithelial cells, and enhances matrix metalloproteinases production, therefore playing a fundamental role in tissue remodeling. Moreover, HGF also stimulates blood vessel formation and promotes vascularization, a later process in wound healing.

In gingival fibroblasts, thrombin and a specific PAR_1_-activating peptide-induced proliferation through endothelin-1 (ET-1) are involved in drug-induced proliferation of gingival fibroblasts [14]. In this context, an in vitro study [15] demonstrated that thrombin and PAR_1_ agonist induced connective tissue growth factor (CTGF) synthesis and TGF-*β*1 activation in gingival fibroblasts. TGF-*β*1and CTGF are proteins that regulate many biological effects, such as cell adhesion, migration, differentiation, proliferation, extracellular matrix production, angiogenesis, and wound healing. As well as ET-1, it is suggested that its overexpression may be involved in gingival overgrowth.

Moreover, Rohani et al. [16] showed that activation of PAR_1_ by thrombin in gingival epithelial cells leads to induction of chemokines which are important chemo-attractants for neutrophils and have a role in wound healing.

Thrombin exerts multiple effects upon osteoblasts including stimulating proliferation and inhibiting osteoblast differentiation and apoptosis. Some of these effects such as synthesis and secretion of growth factors and cytokines are believed to be mediated through PAR activation. In fact, Pagel et al. [51] demonstrated that thrombin induced TGF-1*β*, cyclooxygenase-2, tenascin C, fibroblast growth factors 1 and 2, connective tissue growth factor, and IL-6 expression in wild-type osteoblasts, but not in PAR_1_ knockout mouse osteoblasts. In addition, PAR_1_-specific activating peptide and thrombin induced release of both prostaglandin E_2_ and IL-6 by osteoblasts, therefore suggesting the receptor participation in the earliest stages of bone healing.

The other evidence that links PAR_1_ action to bone metabolism comes from the fact that in periodontal ligament cells, PAR_1_ activation by thrombin induces the synthesis of osteoprotegerin, which is one of the key molecules that regulate bone homeostasis and prevent osteoclastogenesis [12]. Corroborating with these findings, a recent study found that the activation of PAR_1_ in monocytic cells by plasmin diminished LPS-induced inflammatory osteoclastogenesis and bone resorption by inactivation of nuclear factor kappa beta (NF-*κ*B) [13]. Conversely, Uehara et al. [17] showed that the gingipains Rgp and Kgp synergistically increase the secretion of proinflammatory cytokines such as IL-8 from human monocytic cells via PAR_1_, PAR_2_, and PAR_3_ in combination with Toll-like receptors or NOD agonists (pathogen-associated molecular pattern receptors). This study was the first one to report that gingipains stimulate the secretion of cytokines from monocytic cells through the activation of PARs with synergistic effects by pathogen-associated molecular patterns (PAMPs). In addition, Giacaman et al. [52] have showed that selective cleavage of PAR_1_ on oral epithelial cells by the gingipain Rgp upregulates expression of the proinflammatory cytokines IL-1*α*, IL-1*β*, IL-6, and tumor necrosis factor alpha (TNF-*α*).

Interestingly, gingipains-R (RgpB and HRgpA) were also shown to activate PAR_1_ in platelets leading to platelet aggregation [18]. This mechanism may explain the biological plausibility of the association between periodontitis and cardiovascular disease and deserves further clarification by future studies.

Taken together, the results from these in vitro studies show that PAR_1_ is associated with both proinflammatory and reparative processes in the periodontium. However, Wong et al. [53] found no difference between PAR_1_^+/+^ and PAR_1_ knockout mice with regard to alveolar bone loss in a *Porphyromonas gingivalis-*induced periodontal disease model, indicating that this receptor does not play a pivotal role in the progression of experimental periodontitis. Most recently, it was shown by Spolidorio et al. [54] that parstatin, a 41-amino acid peptide released upon PAR_1_ activation, has potential anti-inflammatory effects since it decreases inflammatory cell infiltration, myeloperoxidase (MPO) activity, and proinflammatory mediators' levels, including IL-1*β*, IL-6, and TNF-*α* in gingival tissues of rats subjected to experimental periodontal disease.

Furthermore, a recent study by da Silva et al. [47] has suggested the first clinical evidence of the association of PAR_1_ with periodontal repair. The authors demonstrated that PAR_1_ expression was downregulated in chronic periodontitis patients and inversely correlated to gingival crevicular fluid levels of IL-6, IL-8, TNF-*α*, IFN-*γ*, and MMP-2. In addition, periodontal therapy resulted in PAR_1_ overexpression by epithelial and immune cells from the gingival crevicular fluid, therefore suggesting the importance of PAR_1_ mediating the known anabolic actions of thrombin in the periodontium.

## 4. Biological Effects of PAR_2_ Activation in Periodontal Cells and Tissues

PAR_2_ acts as a “sensor” of bacterial and host proteinases and modulates host immune defense playing a role in the host alarm system [55, 56]. PAR_2_ has been localized in many cell types (Table 3) that can be found in periodontal tissues, including neutrophils, osteoblasts, oral epithelial cells, and human gingival fibroblasts [18, 21, 50, 57]. Gingipains from *Porphyromonas gingivalis*, neutrophil proteinase 3, and mast cell tryptase are the agonists that can possibly be found at the periodontal environment and that have already been studied for their ability to activate PAR_2_.

Gingipains have been shown to activate PAR_2_ in immunoinflammatory cells that play important roles in periodontal disease development. For instance, PAR_2_ activation by RgpB leads to neutrophil activation as indicated by increased intracellular calcium concentrations [57]. In addition, gingipains (Rgps and Kgp) may activate PARs (PAR_1_, PAR_2_, and PAR_3_) in monocytic cells increasing production of IL-6, IL-8, and monocyte chemoattractant protein- (MCP-) 1 [17]. Furthermore, Yun et al. [58] showed that RgpA activated the proteinase-activated receptors and induced T-cell activation. Taken together, these findings suggest that *Porphyromonas gingivalis*, through its gingipains, makes use of the host cell PAR_2_ to exacerbate inflammation during chronic periodontal disease.

One of the most important mechanical barriers that the bacteria encounter to invade the periodontium is the epithelial tissue. Besides the physical barrier, the epithelial tissues have also the ability to produce antimicrobial peptides such as the *β*-defensins (hBD). Interestingly, a study by Chung et al. [59] showed that gingipains may also play a protective role by increasing hBD-2 expression in gingival epithelial cells partially through PAR_2_ receptor signaling pathway. In addition, Pereira et al. [60] have showed that in subjects with chronic periodontitis there are significantly higher levels of *Porphyromonas gingivalis* associated with increased salivary hBD-2 levels and gingival crevicular fluid PAR_2_ mRNA expression than in healthy subjects and that periodontal treatment decreases both hBD-2 levels and PAR_2_ expression. On the other hand, gingipains have also been shown to activate PAR_2_ on oral epithelial cells leading to the production of proinflammatory mediators, such as IL-6 [18] and IL-8 [17] that could result in periodontal tissue breakdown. Moreover, Giacaman et al. [52] suggested that gingipains Rgp and Kgp may cleave and activate PAR_2_ in oral keratinocytes upregulating the expression of IL-1*α*, IL-1*β*, IL-6, and TNF-*α*. Furthermore, a recent study by Tada et al. [61] found that the expression of IL-33, a cytokine that augments Th2 cytokine-mediated inflammatory responses, is increased during *Porphyromonas gingivalis* infection in human gingival epithelial cells via PAR_2_ through gingipain-dependent activation.

As the bacteria challenge increases, an enhanced permeability of the small blood vessels of the subgingival plexus occurs resulting in an increased neutrophil migration through the junctional epithelium and into the gingival sulcus. Interestingly, activated neutrophils may secrete a proteinase (neutrophil proteinase 3) which was shown to activate human oral epithelial cells through PAR_2_, inducing IL-8 and monocyte chemoattractant protein-1 production [17].

Increased levels of proinflammatory mediators and pathogenic bacteria in the soft tissues may lead to the disruption of the epithelial tissue, which in turn facilitates the access of bacteria and their products to the subepithelial connective tissue. The exposure of the residing periodontal connective tissue cells to the bacterial agents may transform them into major participants in the pathophysiological process of periodontal tissue destruction. The dominant cell type in periodontal connective tissue is the fibroblast. Interestingly, Uehara et al. [50] demonstrated that human gingival fibroblasts express PAR_2_ and that its activation by a synthetic PAR_2_ agonist peptide (SLIGRL) induces the production of IL-8 which has the ability to selectively stimulate MMP activity, responsible for collagen destruction within periodontitis lesions. *Porphyromonas gingivalis* may exacerbate this process since it was demonstrated that gingipains upregulate PAR_2_ gene expression in human gingival fibroblasts [62].

Abraham et al. [21] demonstrated that PAR_2_ is expressed by osteoblasts and that its activation by a specific synthetic peptide did not show any effect on osteoblast proliferation or differentiation. In addition, in this study, osteoblast-mediated osteoclast bone resorption was also not stimulated by PAR_2_ activation. Furthermore, Smith et al. [63] showed that PAR_2_ activation inhibits expression of receptor activator of nuclear factor kappa-B ligand (RANKL) and suppressed the RANKL : osteoprotegerin ratio in osteoblasts. However, a study by Amiable et al. [64] showed that PAR_2_ activation in osteoarthritis subchondral bone osteoblasts induced a significant upregulation of RANKL and significantly enhanced bone resorptive activity. Interestingly, these findings on the resorptive properties played by PAR_2_ in osteoblasts are in agreement with data reporting the involvement of PAR_2_ activity in periodontitis [6, 30–33, 65].

Accordingly, it has been shown that a selective PAR_2_ agonist (SLIGRL) causes periodontitis in rats through a mechanism involving prostaglandin release and MMP activation [65] and that PAR_2_-knockout mice infected with *Porphyromonas gingivalis* have decreased levels of proinflammatory mediators, such as prostaglandin E_2_, interferon-gamma, IL-1beta, and IL-6, and less alveolar bone loss when compared to wild-type animals [6].

Wong et al. [53] also have shown that less alveolar bone resorption occurred in PAR_2_-knockout mice. In addition, they showed that T-cells from *Porphyromonas gingivalis*-infected PAR_2_^−/−^ mice proliferated less in response to antigen than those from wild-type mice and that T-cells from infected or antigen-immunized PAR_2_-null mice had a significantly different Th1/inflammatory cytokine profile from wild-type cells such as decreased gamma interferon, ILs (IL-2, IL-3, and IL-17), granulocyte-macrophage colony-stimulating factor, and TNF-alpha than wild-type controls. The absence of PAR_2_ therefore appears to substantially decrease T-cell activation and the Th1/inflammatory response. In this study [53], increased numbers of mast cells in the maxillary tissue of infected PAR_2_^+/+^ mice were also shown, indicating that PAR_2_ may also have a role in mast cell differentiation or infiltration into tissues. Thus, activation of PAR_2_ expressed by mast cells by the arginine-specific gingipains from *Porphyromonas gingivalis* may lead to the release of inflammatory mediators that are pivotal to early inflammatory response in chronic periodontitis. It has been shown that activation of PAR_2_ leads to degranulation of mast cells, causing the release of proinflammatory compounds that kill pathogens and upregulate the immune responses. In addition, tryptase, released from the granules of mast cells upon degranulation, may also activate PAR_2_, and therefore, these cells could play a role in periodontitis by causing the activation of the receptor on other cells in the periodontal tissues. Thus, the regulation of such proinflammatory mechanisms in T-cells and mast cells by PAR_2_ suggests a pivotal role in the pathogenesis of the disease (Table 4).

In the gingival crevicular fluid from patients with periodontal disease, there are high levels of proteolytic activity characterized by a mixture of endogenous and exogenous proteinases which may mediate degradation of connective tissue [66]. Among these hydrolytic enzymes in the inflamed periodontal environment, neutrophil proteinase 3, mast cell tryptase, and gingipains have been isolated and are known to activate PAR_2_. In fact, high levels of proteolytic activity derived from both *Porphyromonas gingivalis* and neutrophils are expected to be found in the periodontal pocket of chronic periodontitis, since they are, respectively, the major periodontal pathogen and the predominant cells (approximately 90%).

PAR_2_ has been shown to be expressed by cellular elements found in gingival crevicular fluid, which may include epithelial cells, and leukocytes even in clinically healthy human gingival sulci [30]. In addition, PAR_2_ expression is upregulated in chronic periodontitis patients compared to that in healthy controls (Table 5). Interestingly, PAR_2_ upregulation in the inflamed periodontium is associated with an elevated gingival crevicular fluid trypsin-like activity, probably due to the increased prevalence of *Porphyromonas gingivalis*, and expression of neutrophil proteinase 3 [30].

Importantly, proteinase 3 has been shown to activate oral epithelial cells through PAR_2_ leading to the production of IL-8 and monocyte chemoattractant protein-1 [17]. These findings clearly suggest that proteinase 3 actively participates in PAR_2_-mediated inflammation at periodontal sites.

PAR_2_ activation results in the synthesis of proinflammatory mediators including IL-6, IL-8, Il-1, IFN-*γ*, PGE_2_, and MMP-9 [6, 67, 68] and activates signaling pathways such as those involving mitogen-activated protein kinase and nuclear factor-*κ*B, which potentiates inflammatory responses [69]. Interestingly, proinflammatory mediators such as TNF-alpha and IL1-beta are reported to increase PAR_2_ expression [30]. Accordingly, an increased PAR_2_ expression has been demonstrated in deeper periodontal pockets compared to the expression of the receptor in shallower pockets, and it was associated with significant increased levels of proinflammatory mediators [30]. In addition, periodontal treatment statistically reduces PAR_2_ expression [30, 32]. Furthermore, Fagundes et al. [31] demonstrated that the presence of *Porphyromonas gingivalis* is associated with an elevated expression of PAR_2_ in human chronic periodontitis, thus suggesting that *Porphyromonas gingivalis* may disturb the host inflammatory responses not only by regulating PAR_2_ function but also by enhancing its genetic expression. Taken together, these findings clearly suggest that PAR_2_ overexpression is an essential element in inflammation severity.

Another study by Euzebio Alves et al. [32] showed that PAR_2_-positive staining in gingival crevicular fluid cells was reflective of tissue destruction, and its overexpression was positively associated to inflammatory clinical parameters and to the levels of the proinflammatory mediators IL-6, IL-8, TNF-alpha, MMP-2, MMP-8, hepatocyte growth factor, and vascular endothelial growth factor (VEGF). Another interesting finding from this study was that periodontally healthy sites from chronic periodontitis individuals showed a diminished expression of PAR_2_ mRNA and PAR_2_ protein level, therefore suggesting that PAR_2_ periodontal expression is influenced by the presence of infection and not merely a constitutive characteristic that may favor periodontal inflammation.

## 5. Specific Synthetic Agonists

Selective synthetic peptides, corresponding to the tethered ligand sequences, are able to activate selectively the receptors through direct binding to the body of the receptor without the need of proteolysis [22]. With the exception of PAR_3_, all the other receptors have their selective agonist peptides. PAR_1_, PAR_2_, and PAR_4_ can be nonenzimatically and selectively activated by TFLLR-NH2, SLIGRL-NH2, and GYPGQVNH2, respectively [1, 2]. Since their discovery, selective PAR agonists have been used in order to assess the specific impact of PAR_1_, PAR_2_, and PAR_4_ signaling.

Noteworthy, earlier literature in many instances used the “TRAP” peptide (SFLLRN) to activate “PAR_1_” not realizing that the peptide also coactivates PAR_2_ [36]. Thus, the earlier use of the “TRAP” peptide may have generated results that reflect the coactivation of both PARs 1 and 2. Furthermore, the PAR_2_-activating peptide (SLIGRL-NH2) can in some settings cross-react with the MAS receptor, whereas the use of a more potent 2-furoyl-LIGRLO-amide can prevent this issue [36].

## 6. Recent PAR Antagonists

The newest PAR_1_ antagonist (Vorapaxar) has been used for the treatment of cardiovascular diseases. Vorapaxar acts by blocking the docking of the tethered ligand sequence preventing PAR_1_ activation, hence platelet aggregation [10]. Regarding PAR_2_ blockage, the antagonist GB88 can block PAR_2_ activation by trypsin as well as PAR-activating peptide. GB88 has been shown to inhibit PAR2-induced proinflammatory cytokine release and attenuate inflammation in a rat model of colitis [10]. However, until now, none of these antagonists were studied in periodontics.

## 7. PARs: Drug Targets in Inflammation

Therapies focusing on the inhibition of proteinases or, more specifically, the use of PAR antagonists may constitute an important approach for the modulation of an infectious pathology such as periodontal inflammatory disease.

According to Yun et al. [58], PAR_2_ blockage with the use of antagonists might promote adverse high proteolytic activities in the gingival crevicular fluid. Thus, it seems that the potential side effects that the concept of PAR_2_ blockade encounters do not seem to overcome the beneficial aspects for the treatment of periodontal inflammation.

At a certain point in the inflammatory process, PAR 1 and 2 blockages may be necessary to prevent increased inflammation, whereas at later time points, PAR activation may be required to aid resolution. Further, the use of specific proteinase inhibitors, rather than PARs antagonists, may result in a “dual” blockage inhibiting both the degradation of host molecules and activation of PARs. Golub et al. [70] found that tetracyclines can inhibit tissue collagenolytic activity in in vivo, in vitro, and periodontal pockets presented by subjects with and without diabetes. Castro et al. [33] found that administration of a subantimicrobial dose of doxycycline (SDD) in a rat periodontitis model may result in PAR_2_ modulation through a dual mode, downregulating its gene expression and decreasing its posterior activation by proteinases. Doxyxycline in a subantimicrobial dose is able to inhibit the activity of MMPs and thus reduce the degradation of collagen, fibronectin, and elastin in the periodontal tissues [71], and its clinical use in the modulation of the immunoinflammatory host response as coadjuvant to periodontal conventional therapy is approved by the Food and Drug Administration since 1998. Moreover, in periodontal inflammation, the inhibition of MMPs by drugs from the tetracycline family may not only attenuate the activation of PARs by MMPs but also can prevent extracellular matrix remodeling which can sequester cell-regulating polypeptide that in turn can act together with the PAR- promoting fibrosis [2]. However, additional studies are necessary to confirm the clinical benefits of tetracycline family and other highly specific inhibitors on PAR-mediated periodontal inflammation.

## 8. Concluding Remarks

PARs together with Toll-like receptors and NOD-like receptors are pattern recognition receptors that contribute to innate immunity. A new paradigm in microbial pathogenicity has been established in which bacterial proteinases manipulate host cell functions through PAR activation. An uncontrolled PAR activation will certainly result in a disruptive local inflammatory response, which can benefit the periodontal microbial community.

The in vitro studies described here highlight the differential actions of PARs on periodontal cells, suggesting that PARs, like other components of the innate immunity, act as a double-edged sword with both protective and destructive responses. However, in periodontal tissues, PAR_2_ seems to be upregulated during inflammation, where it is believed to be activated by bacterial and host proteinases, whereas PAR_1_ is upregulated during periodontal tissue repair. This counterregulation of PARs actions was also demonstrated by Xue et al. [72] in rheumatoid arthritis synovial fibroblasts, where PAR_2_ activation was associated with an elevated TNF-alpha release and PAR_1_ activation prevented the release of proinflammatory cytokines. Interestingly, da Silva et al. [47] showed that PAR_1_ overexpression after periodontal treatment was inversely correlated to PAR_2_ expression in gingival crevicular fluid cells. In addition, Zhang et al. [73] observed that PAR_2_ is upregulated whereas PAR_1_ is downregulated in human gingival epithelial cells during *Porphyromonas gingivalis* infection. Understanding the mechanisms that keep their functions in balance can bring new knowledge on the role of PARs in the development and treatment of periodontal inflammation.

## Figures and Tables

**Table 1:** Potential activators of PARs in the periodontium.

Protease	Protease-3	Thrombin	Plasmin	Tryptase	MMP-1 & MMP-13	Gingipains	Dentilisin	Aa protease
Origin	Neutrophils	Fibrin clot	Fibrin clot	Mast cells	Monocytes/macrophages	*Porphyromonas gingivalis*	*Treponema denticola*	*Aggregatibacter actinomycetemcomitans*
Type	Serine protease	Serine protease	Serine protease	Chymotrypsin-like protease	Collagenases	Cysteine proteases (i) Arginine (HRgpA and RgpB) (ii) Lysine (Kgp)	Chymotrypsin-like enzyme	Arginine and lysine proteases
PAR interaction	PAR_2_ activation	PAR_1_ activation PAR_3_ activation PAR_4_ activation	PAR_1_ activation	PAR_2_ activation	PAR_1_ activation	PAR_1_ activation PAR_2_ activation	PAR_2_ disarming	PAR_2_ activation

PAR: protease activated receptor; MMP: matrix metalloproteinase; Aa: *Aggregatibacter actinomycetemcomitans*.

**Table 2:** Biological effects of PAR_1_ activation in periodontal cells.

PAR_1_	Periodontal destruction	Periodontal repair/protection
Oral epithelial cells	↑ IL-1*α*, IL-1*β*, IL-6, TNF*α* [52] ↑ CXCL 5 [16]	
Gingival fibroblasts		↑ HGF [50] ↑ endothelin-1 [14]
Osteoblasts	↑ COX-2 [51] ↑ IL-6 [51] ↑ PGE_2_ [51]	↑ TGF-*β* [51] ↑ FGF-1/FGF-2 [51] ↑ CTFG [51]
Periodontal ligament cells		↑ osteoprotegerin [12]
Monocytic cells	↑ IL-8 [17]	

PAR: protease-activated receptor; IL: interleukin; TNF: tumor necrosis factor; CXCL: C-X-C motif chemokine; COX: cyclooxygenase; PGE: prostaglandin E; HGF: hepatocyte growth factor; TGF: transforming growth factor; FGF: fibroblast growth factor; CTFG: connective tissue growth factor.

**Table 3:** Biological effects of PAR_2_ activation in periodontal cells.

PAR_2_	Periodontal destruction	Periodontal repair/protection
Epithelial cells	↑ IL-1*α*, IL-1*β*, IL-6, IL-8, TNF*α* [4, 47] ↑ MCP-1 [17] ↑ IL-33 [61]	↑ *β*-defensin 2 [59]
Fibroblasts	↑ IL-8 [62]	
Osteoblasts	↑ RANKL [64]	↓ RANKL [63] ↓ RANKL : osteoprotegerin ratio [63]
Monocytic cells	↑ IL-6, IL-8, MCP-1 [17]	

IL: interleukin; MCP: monocyte chemoattractant protein; RANKL: receptor activator of nuclear factor kappa-B ligand.

**Table 4:** In vitro studies on PAR_1_ and PAR_2_ activation associated with periodontal tissue metabolism.

Author(s)/year	In vivo model	PAR	Experimental groups	Biological effect(s)	Mechanism(s) involved
Holzhausen et al. [65]	Male Wistar rats subjected to ligature-induced periodontitis (right mandibular first molar)	PAR_2_	(i) Ligature + saline, *n* = 32 (ii) Ligature + SLIGRL-NH_2_ (agonist peptide), *n* = 32 (iii) Ligature + LRGILS-NH_2_ (control peptide), *n* = 32 (iv) Sham + saline, *n* = 32 (v) Sham + SLIGRL-NH_2_, *n* = 32 (vi) Sham + LRGILS-NH_2_, *n* = 32 Sacrifice at 3, 7, 15, and 30 days	PAR_2_ agonist induced alveolar bone loss and granulocyte infiltration and exacerbated ligature-induced periodontitis.	Upregulation of COX-1, COX-2, MMP-2, and MMP-9.
Holzhausen et al. [6]	PAR2-deficient mice subjected to *P. gingivalis* oral infection	PAR_2_	(i) PAR_2_^−/−^ mice + *P. gingivalis* oral infection, *n* = 20 (ii) PAR_2_ WT mice + *P. gingivalis*, *n* = 20 Sacrifice at 42 and 60 days	PAR_2_^−/−^ mice showed less alveolar bone loss compared to WT mice.	PAR_2_ activation in the presence of *P. gingivalis* infection increased inflammatory cell infiltration, prostaglandin-E_2_, IFN-*γ*, IL-6, and IL-1*β* levels.
Wong et al. [53]	PAR2-deficient mice subjected to *P. gingivalis* oral infection	PAR_2_	(i) PAR_2_^−/−^ mice + *P. gingivalis* oral infection, *n* = 24 (ii) PAR_2_ WT mice + *P. gingivalis*, *n* = 24 Sacrifice at 30 days	PAR_2_^−/−^ mice showed less exposed root surface and less alveolar bone eroded surface compared to WT mice.	PAR_2_-deficient mice showed decreased infiltration of mast cells in the periodontal tissues and impaired T-cell immune responses (decreased activation and Th1/inflammatory response).
	PAR1-deficient mice subjected to *P. gingivalis* oral infection	PAR_1_	(i) PAR_1_^−/−^ mice + *P. gingivalis* oral infection, *n* = 24 (ii) PAR_1_ WT mice + *P. gingivalis*, *n* = 24 Sacrifice at 30 days	No difference between PAR_1_^−/−^ and PAR_1_ WT mice with regard to the exposed root surface.	
Holzhausen et al. [46]	Male Wistar rats subjected to ligature-induced periodontitis (right mandibular first molar)	PAR_2_	(i) Control group: daily i.p. administration of saline, *n* = 20 (ii) Ligature group: ligature placement and daily i.p. administration of saline, *n* = 20 (iii) Nafamostat mesilate (NM) group: NM (0.1 mg/kg/day, i.p.) a potent tryptase inhibitor, *n* = 20 (iv) NM + ligature group: ligature and daily i.p. NM (0.1 mg/kg/day), *n* = 20 Sacrifice at 7 and 14 days	Tryptase inhibition decreased alveolar bone loss, MPO, and total proteolytic activity in animals subjected to ligature-induced periodontitis.	Tryptase inhibition led to a 1.6-fold decrease in gingival PAR_2_ expression.
Spolidorio et al. [54]	Male Wistar rats subjected to ligature-induced periodontitis (upper second molars)	PAR_1_	(i) Ligature + intraoral 3 *μ*g parstatin, *n* = 16 (ii) Ligature + intraoral PBS, *n* = 16 (iii) Sham, *n* = 8 Sacrifice at 8 and 15 days	Parstatin (peptide released upon PAR_1_ activation) prevented periodontal tissue breakdown.	Parstatin suppressed inflammatory cell infiltration and decreased MPO, IL-1*β*, TNF-*α*, and IL-6.
Castro et al. [33]	Male Wistar rats subjected to ligature-induced periodontitis (mandibular first molars)	PAR_2_	(i) No ligature and no treatment, *n* = 20 (ii) Ligature + placebo (0,9% NaCl solution), *n* = 20 (iii) Ligature + 5 mg subantimicrobial dose of doxycycline (SDD) by daily gavage, *n* = 20 Sacrifice at 3 and 15 days	SDD downregulated alveolar bone loss.	Downregulation of PAR_2_, IL-17, IL-1*β*, and TNF-*α*.

PAR: protease-activated receptor; COX: cyclooxygenase; MMP: matrix metalloproteinase; *P. gingivalis*: *Porphyromonas gingivalis*; WT: wild-type mice; IL: interleukin; IFN-*γ*: interferon gamma; MPO: myeloperoxidase; PBS: phosphate-buffered saline; TNF: tumor necrosis factor.

**Table 5:** Human studies on PAR_1_ and PAR_2_ activation associated with periodontal tissue metabolism.

	Groups	Main findings
Holzhausen et al. [30]	(i) Controls: 40 subjects (ii) Chronic periodontitis with moderate destruction (3 < PPD ≤ 6 mm; CAL ≤ 4 mm), *n* = 40 (iii) Chronic periodontitis with advanced destruction (PPD > 6 mm; CAL > 4 mm), *n* = 40	(i) Chronic periodontitis patients showed higher PAR_2_ mRNA expression and increased levels of IL-1*α*, IL-6, IL-8, TNF-*α*, total proteolytic activity, *P. gingivalis* prevalence, and proteinase 3 mRNA expression compared to controls. (ii) Periodontal treatment decreased PAR_2_ mRNA expression.
Fagundes et al. [31]	(i) Moderate chronic periodontitis (3 < PPD ≤ 6 mm; 3 < CAL ≤ 6 mm), *n* = 35	*P. gingivalis* presence was associated with the following: (i) higher levels of IL-1*α*, IL-6, and TNF-*α*, (ii) higher proteolytic activity, (iii) higher PAR2 mRNA expression.
Euzebio Alves et al. [32]	(i) Controls: 31 subjects (ii) Moderate chronic periodontitis (3 < PPD ≤ 6 mm; CAL ≤ 4 mm), *n* = 31	Chronic periodontitis patients showed overexpression of PAR_2_, IL-6, IL-8, TNF-*α*, MMP-2, MMP-8, HGF, VEGF, gingipain, and proteinase 3 and decreased levels of dentilisin, SLPI, and elafin. Periodontal treatment decreased PAR_2_ expression.
da Silva et al. [47]	(i) Controls: 37 subjects (ii) Moderate chronic periodontitis (3 < PPD ≤ 6 mm; CAL ≤ 4 mm), *n* = 38	Periodontal treatment resulted in PAR_1_ overexpression, which was inversely correlated with PAR_2_ expression and gingival crevicular fluid levels of IL-6, IL-8, TNF-*α*, IFN-*γ*, and MMP.

PAR: protease activated receptor; IL: interleukin; *P*. *gingivalis*: *Porphyromonas gingivalis*; PPD: probing pocket depth; CAL: clinical attachment level; TNF: tumor necrosis factor; P3: neutrophil serine protease 3; MMP: matrix metalloproteinase; HGF: hepatocyte growth factor; VEGF: vascular endothelial growth factor; SLPI: secretory leukocyte protease inhibitor; GCF: gingival crevicular fluid; IFN-*γ*: interferon gamma.
